# Clade homogeneity and low rate of delta virus despite hyperendemicity of hepatitis B virus in Ethiopia

**DOI:** 10.1186/s12985-017-0844-z

**Published:** 2017-09-12

**Authors:** Yeshambel Belyhun, Uwe Gerd Liebert, Melanie Maier

**Affiliations:** 1 Institute of Virology, Medical Faculty, Leipzig University, Johannisallee 30, 04103 Leipzig, Germany; 2 School of Biomedical and Laboratory Sciences, College of Medicine and Health Sciences, University of Gondar, Gondar, Ethiopia

**Keywords:** HDV, Genotype, Clade homogeneity, Ethiopia

## Abstract

**Background:**

Although hepatitis B virus (HBV) is hyperendemic and heterogeneous in its genetic diversity in Ethiopia, little is known about hepatitis D virus (HDV) circulating genotypes and molecular diversity.

**Methods:**

A total of 321 hepatitis B surface antigen (HBsAg) positives (125 HIV co-infected, 102 liver disease patients and 94 blood donors) were screened for anti-HDV antibody. The anti-HDV positive sera were subjected to Real time PCR for HDV-RNA confirmation. The non coding genome region (spanning from 467 to 834 nucleotides) commonly used for HDV genotyping as well as complete HDV genome were sequenced for genotyping and molecular analysis.

**Results:**

The anti-HDV antibody was found to be 3.2% (3) in blood donors, 8.0% (10) in HIV co-infected individuals and 12.7% (13) in liver disease patients. None of the HIV co-infected patients who revealed HBV lamivudine (3TC) resistance at tyrosine-methionine/isoleucine-aspartate-aspartate (YM(I)DD) reverse transcriptase (RT) motif with concomitant vaccine escape gene mutants was positive for anti-HDV antibody. The HDV viremia rate was 33.3%, 30.0% and 23.1% in respect to the above study groups. All the six isolates sequenced were phylogenetically classified as HDV genotype 1 (HDV-1) and grouped into two monophyletic clusters. Amino acid (aa) residues analysis of clathrin heavy chain (CHC) domain and the isoprenylation signal site (Py) at 19 carboxyl (C)-terminal amino acids (aa 196–214) and the HDV RNA binding domain (aa 79–107) were highly conserved and showed a very little nucleotide variations. All the sequenced isolates showed serine at amino acid position 202. The RNA editing targets of the anti-genomic HDV RNA (nt1012) and its corresponding genomic RNA (nt 580) showed nucleotides A and C, respectively.

**Conclusions:**

The low seroprevalence and viraemic rates of HDV in particular during HIV-confection might be highly affected by HBV drug resistance selected HBsAg mutant variants in this setting, although HDV-1 sequences analysis revealed clade homogeneity and highly conserved structural and functional domains. Thus, the potential role of HBV drug resistance associated polymerase mutations and concomitant HBsAg protein variability on HDV viral assembly, secretion and infectivity needs further investigation.

## Background

HDV is a defective RNA virus and an obligate satellite of HBV that infects humans either concomitantly with HBV or after HBV infection [1]. Its genome is a circular, negative sense, and single-stranded RNA of approximately 1.7 kb in length [1].The HDV anti-genome contains a unique open reading frame that encodes the small (sHDAg) and large (LHDAg) hepatitis delta antigens from the same reading frame with amino acids length of 195 and 214, respectively [1, 2]. HDV prevalence and geographic distribution is not uniform and epidemiological information from most parts of the world is still lacking [3]. However, it is generally considered to be endemic in Africa, Asia, Latin America, Mediterranean regions and Eastern Europe [3, 4]. In contrast, HDV prevalence is still low in the developed western countries [4, 5] despite a changing pattern due to immigration from the endemic regions [4, 5]. From the globally estimated 350 million HBsAg carriers, 15 to 20 million people are infected with HDV which is associated with a severe form of viral hepatitis [6]. Although the mechanism of HDV pathogenesis has not been fully explained [3], the severity of liver disease due to HDV varies by the nature of its co-or super-infection with HBV and as well the genotypes of both viruses [7].

Clinically, the co-infection of HBV with HDV leads to acute hepatitis, but subsequent chronic infection is rare, whereas the super-infection of HDV in HBV carriers typically induces a severe form of hepatitis and causes chronic infection compared to HBV mono-infection [3]. The variation in clinical severity of HDV may also be determined by host and viral genetic factors and the geographical discrepancies by the disease severity is usually associated with genotypes of HDV [8]. To date, eight distinct HDV clades or genotypes are recognized based on their sequence homology and are labeled as HDV-1 to HDV-8 [9]. Still, to characterize more HDV isolates from different parts of the world have a crucial virological and clinical meaning [8]. In addition, whether combinations of HBV genotypes with HDV genotypes cause varying clinical outcomes, remains to be explored [8, 10].

Moreover, compared with HBV mono-infection, human immunodeficiency virus (HIV)-HBV co-infections may lead to accelerated hepatic disease progression with higher rates of liver cirrhosis and liver-related mortality and complicate treatment possibilities [11]. Notably, rate of HDV infection has been increasing among HBV-HIV-co-infected patients [10]. Nevertheless, there is scarce information about HDV prevalence, virological profile and its natural history among HIV patients [7] especially in areas where HDV is endemic and routine HBV immunization is not common [12]. In particular, while HBV seroprevalence is very high across African countries [13], little information is available on the HDV origin, circulation, and molecular diversity. Similarly, in Ethiopia where HBV remains unchecked for clinically suspected liver disease patients and HIV co-infected individuals coupled with absence of HBV vaccination coverage for the adult population, molecular epidemiology and clinical spectrum of liver disease due to HDV viral origin are unknown. So far, there is no molecular information about HDV from Ethiopia. This study was, therefore, aimed to determine molecular epidemiology and genetic diversity of HDV isolated from HBV-HIV co-infected and HBV mono-infected Ethiopians.

## Methods

### Patients/study participants

In 2013, a total of 4105 study samples were collected from HIV infected individuals (*n* = 2133), blood donors (*n* = 1720) and chronic liver disease (CLD) patients (*n* = 252) in northwest Ethiopia. Among this total study population, 340 HBsAg positive sera were studied before to determine clinically important HBV drug resistant and immune (vaccine) escape HBsAg gene variants among HIV co-infected and HBV mono-infected Ethiopians [14]. In this cross-sectional study, from the above well characterized HBsAg positive sera, 321 of them were screened for anti-HDV antibody with the proportions of blood donors (*n* = 94), CLD patients (*n* = 102) and HIV co-infected patients (*n* = 125). The demographic data and clinical characteristics of the study subjects screened for HDV were collected during blood sample collection as described earlier [14].

### HDV antibody screening

A total of 321 HBsAg positives sera was tested for qualitative total anti-HDV antibody (IgG-Ab) using commercially available kit ETI-AB-DELTAK-2 (DiaSorin S.P.A., Saluggia, Italy). The sera were also characterized before [14] for the status of HIV and other hepatitis viruses such as HBV and HCV.

### HDV RNA isolation, quantification and short fragment amplification

HDV RNA was extracted using 200 μl sera from 26 anti-HDV antibody positives cases applying MagNA Pure 96 DNA and Viral NA Small Volume kit on the MagNA Pure 96 Instrument (Roche Diagnostics, Mannheim, Germany). Reverse transcription-Real-Time PCR (RT-rt. PCR) was done for the partial fragment non coding genome region with a spanning of 467–834 nucleotides that commonly used for HDV genotyping [15]. In brief, 5 μl of extracted RNA was heat-incubated at 70 °C for 5 min to diminish secondary structures. The RNA was then reverse transcribed using Super Script III reverse transcriptase enzyme (Invitrogen, Karlsruhe, Germany) and Random-Hexamer-Primers (Roche Applied Science, Mannheim, Germany). The RT was performed in a final volume of 20 μl with 4 μl of 5× First strand buffer, 0.1 μl of dithiothreitol (0.1 mol/l), 0.75 μl random Hexames primers (400 ng/μl), 3 μl of dNTP’s (2.5 mmol/l) and 1 μl RNase-inhibitor (40 U/μl). The final RT was performed for 5 min at 25 °C, followed by 50 °C for 35 min and heat activated for 2 min at 95 °C. Two microliters of cDNA were subsequently applied to Real-Time-PCR with PlatinumTaq-Polymerase (Invitrogen, Karlsruhe, Germany) on a LightCycler 2.0 instrument (Roche Diagnostics, Mannheim, Germany) in a 20 μl reaction mixture containing 2.0 μl of 5× Buffer, 1.6 μl of MgCl_2_ (50 mM) BSA (2 mg/ml), dNTP, 0.4 μl of PlatinumTaq Polymerase (5 U/μl), 10 μM of primer HD10 (5′-AGT GAG GCT TAT CCC GG-3′) and primer HD11 (5′-CTC GGA TGG CTA AGG GAG-3′), 12 μM of fluorescein labelled locked nucleic acid probe HD LNA (5′-AG + CCT + CCT + CG + CTGGCGCC-FL-3′) and 4 μM of modified HDV1 light cycler red (LCR) 640 probe (5′- LCRed640-GCTGGGCAACATTCCGAGG-ph–3′). The PCR temperature profiles was as follows: denaturation at 95 °C for 5 min, followed by 15 touch-down-cycles of 95° denaturation for 5 s, annealing at 62–48 °C with 1° reduction each cycle, and elongation at 72 °C for 10 s without measurement and followed by 35 cycles with annealing at 48° and measurement at 640 nm.

### HDV full genome amplification and sequencing

The complete genome amplification of six patients’ HDV RNA positive samples was performed. The need for full genome characterization was necessitated since the above partial fragment (368 nt length) sequences of all the six isolates were genotyped as HDV-1. To produce two partially overlapping segments covering the entire HDV genome (≈ 1670 nt), two sets of primers from Çelik et al. [8] were used with certain modifications. The forward (5-GCG GGC CGG CTACTC TTC TTT C-3′; nt 1160–1180) and the reverse primers (5′-CTA GCC CCG TTG CTT TCT TTG CTT T-3′; nt 410–130) were used for the first fragment amplification covering 950 bp. The other set of primes (forward: 5′-ACC TCCAGA GGA CCC CTT CAG CGA A-3′; nt 300–320 and reverse: 5′-GAG GGA GCT CCC CCG GCG AAGAG-3′; nt 1580–1600) were used for the second fragment amplification covering 1100 bp. The RT reaction was performed at 50 °C incubation using Super Script III reverse transcriptase enzyme (Invitrogen, Karlsruhe, Germany) for 1 h in a total volume of 20 μl RT mixture of 5× First strand buffer, 10 mM dNTPmix (Roche Applied Science, Mannheim, Germany), 0.1 M DTT, 10 μM reverse primers, 40 U/μl Fermentas RiboLock and 200 U/μl and 5 μl template RNA. The PCR were performed with 5 μl of cDNA mixed with 10 μM of each primer, 10 mM dNTPmix, 50 mM MgCl_2_, 5 U/μl Taq DNA polymerase (Promega, Madison, WI, USA) and 10× buffer to a final volume of 50 μl. Then the PCR was performed at 94 °C for 1.5 min followed by 35 cycles at 94 °C for 30s, 55 °C for 30s and 72 °C for 1.5 min and a final extension for 3 min at 72 °C. The RT and PCR reaction components, concentrations and conditions used for both fragments were similar. The amplified PCR products for both non-coding partial genome region and the full genome were gel extracted from 1.5% agarose gel and purified using the Wizard SV Gel and PCR Clean-Up System (Promega, Mannheim, Germany). Strict precautions were followed to avoid cross contamination and appropriate positive and negative controls were included during DNA extraction and PCR amplification steps. Finally, the sequence products were subjected to direct sequencing using the ABI Prism BigDye Terminator cycle sequencing reaction kit (Applied Biosystems, Foster City, CA, USA) using ABI Prism 3500 Genetic Analyzer.

### Phylogenetic and sequence analysis

All raw sequences were manually edited and aligned using Geneious software version 6.2.1 (http://www.geneious.com). Neighbor-joining phylogenetic trees and genetic distances were calculated using MEGA5 software (www.megasoftware.net) with Kimura 2-parameter, pair wise deletion option, and 1000 bootstrap replicates. Genotyping was independently performed on the basis of phylogenetic relationship by taking the short fragment non-coding genome region (368pb) and the full HDV genome sequences along with representative reference sequences from different HDV genotypes of 1 to 8 retrieved from the GenBank. Alignment of both the nucleotide sequences and the amino acid residues of the current isolates that encode 19 carboxyl (C)-terminal amino acid (aa 196–214) residues of LHDAg depicting the clathrin heavy chain (CHC) interacting domain (includes clathrin box-binding domain (CBD) (aa 199–203), the isoprenylation (Py) signal site (aa 211–214)) were compared with sequences retrieved from the GenBank. Moreover, nucleotide substitutions variations on the poly(A) signal (nt 946–952), and the RNA editing targets of the anti-genomic HDV RNA (nt 1012) and its corresponding genomic RNA (nt 580) were examined for nucleotide substitution variations. The acetylation (Ac) and phosphorylation (Pi) sites, RNA binding domain (aa 79–107) and the domains of arginine-rich motifs (ARMs) (aa 137–144) of sHDAg were also analysed for possible nucleotide and amino acid residue variations. The nucleotide sequences of both non-coding short fragment genome region and the full HDV genome region used in this study are available in the GenBank/EMBL/DDBJ data bases with an accession numbers from KY463671 to KY463682.

### Statistical analysis

Statistical analysis was performed by using GraphPad Prism Software version 5.01, 2007. Non-parametric data were compared by using the Mann**-**Whitney U test and categorical data were compared by Chi-Square test. A *P*-value of less than 0.05 considered to be statistically significant.

## Results

### Patients/Clients

The anti-HDV positive patients/ clients median interquartile (IQR) age was 38 (30–48) years and their demographic, clinical and virological characteristics are presented in Table 1.

**Table 1:** Patient/client specific clinical and virological characteristics of anti-HDV antibody positives (*n* = 26) and those with HDV full genome sequenced (*n* = 6)

Study groups	Lab. Code	Age/sex	Virological characteristics							Clinical characteristics
			Anti-HCV	HIV status	HBV viral load	HBeAg	HBV genotype	HDV viral load	HDV genotype	
HIV co-infected	**ETH3790**	**34/M**	**Neg**	**Pos**	**3.62**	**Neg** ^**a**^	**A1**	**9.67 × 10** ^**6**^	**I**	**WHO stage I**
	**ETH2170**	**36/F**	**Pos**	**Pos**	**Un**	**Pos**	**–**	**2.27 × 10** ^**5**^	**I**	**WHO stage IV**
	**ETH2280**	**33/M**	**Neg**	**Pos**	**Un**	**Neg**	**–**	**7.08 × 10** ^**7**^	**I**	**WHO stage I**
	ETH1480	37/F	Neg	Pos	2.72	Neg	D2	–	–	WHO stage II
	ETH1570	57/F	Neg	Pos	8.93	Neg	A1	–	–	WHO stage I
	ETH4500	30/F	Pos	Pos	3.08	Neg	D4	–	–	WHO stage II
	ETH1470	31/M	Pos	Pos	0.48	Neg	–	–	–	WHO stage I
	ETH2590	38/F	Neg	Pos	Un	Neg	–	–	–	WHO stage I
	ETH2570	46/M	Neg	Pos	Un	Neg	–	–	–	WHO stage I
	ETH2150	49/F	Neg	Pos	Un	Neg	–	–	–	WHO stage I
CLD patients	**ETH4060**	**60/M**	**Neg**	**Neg**	**6.34**	**Neg**	**A1**	**3.01 × 10** ^**5**^	**I**	**Liver cirrhosis**
	**ETH4100**	**33/M**	**Neg**	**Neg**	**3.15**	**Neg**	**A1**	**2.28 × 10** ^**5**^	**I**	**HCC**
	ETH3020	21/F	Neg	Pos	6.90	Pos	A1	–	–	
	ETH3700	25/M	Neg	Neg	7.60	Pos	A1	–	–	Liver ascites + cirrhosis^a^
	ETH4070	23/M	Neg	Neg	4.04	Neg	D3	–	–	
	ETH5730	45/M	Neg	Neg	4.47	Neg	D2	–	–	Chronic hepatitis^b^
	ETH3080	38/F	Neg	Neg	0.60	Nd	–	–	–	Chronic hepatitis^c^
	ETH3120	64/M	Neg	Neg	6.81	Pos	A1	–	–	
	ETH3190	45/M	Neg	Neg	1.56	Neg	–	–	–	Chronic hepatitis^d^
	ETH3320	52/M	Neg	Neg	1.00	Nd	–	–	–	Liver cirrhosis^a^
	ETH3360	22/M	Neg	Neg	0.78	Neg	–	–	–	
	ETH4390	27/M	Pos	Neg	2.01	Neg	–	–	–	
	ETH4380	28/M	Pos	Neg	Un	Neg	–	2.41 × 10^2^	–	Chronic hepatitis^e^
Blood donors	**ETH2056**	**47/M**	**Neg**	**Neg**	**8.39**	**Neg**	**D2**	**6.55 × 10** ^**5**^	**I**	**–**
	ETH5660	55/M	Neg	Neg	0.78	Neg^a^	–	–	–	–
	ETH5650	38/M	Pos	Neg	Un	Nd	–	–	–	–

Highlighted in bold rows indicate study subjects of their HDV isolate was sequenced. *WHO* World Health Organization HIV infection disease staging, ^a^Both HBeAg and HBeAb tests showed non-reactive, ^b^Complicated with schistosomiasis and Hookworm infections, ^c^Complicated with *H.pylori* infections, ^d^Diagnosed for hepatosplenic and hepatosplenomegaly associated schistosomiasis, ^e^ Complicated with *Ascaris*, Hookworm and *P.falciparum* infections. Note: Chronic liver disease (CLD) patients with no specified clinical characteristics were classified as ‘undefined’ liver sign and symptoms. Un-undetected. Nd-not done (insufficient sample). HCC-Hepatocellular carcinoma

Clinically, three of the six sequenced isolates were from HIV-HBV-HDV triple co-infected patients who were on their HIV ART follow up for 2, 7.7 and 8.1 years whereby isolates ETH3790 and ETH2280 were WHO stage I and the other isolate ETH2170 was stage IV (Table 1). The two patients with isolates ETH4060 and ETH4100 were clinically diagnosed for liver diseases; isolate ETH4060 with liver cirrhosis (LC) and ETH4100 with hepatocellular carcinoma (HCC) (Table 1). The ETH20560 isolate was from an apparently healthy blood donor (Table 1).

### Virological characteristics

**Fig. 1:**
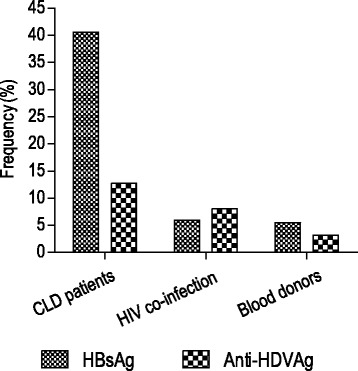
The anti-HDV seroprevalence among HBsAg positives samples respective to each study groups

The anti-HDV prevalence among HBsAg positive samples ranges from 3.2% (3) in blood donors, 8.0% (10) in HBV-HIV co-infected individuals to 12.7% (13) in liver disease patients. Interestingly, none of the HBV-HIV co-infected patients who were characterized by YM(I)DD motif 3TC resistance gene mutations selected vaccine escape HBsAg gene variants (sE164D/sI195M/sW196*) [14] were positive for anti-HDV antibody. The viral RNA positivity rate was 33.3% (1), 30.0% (3) and 23.1% (3) from blood donors, HBV-HIV co-infected and CLD patients, respectively (Fig. 1).

Six out of the seven sera with detectable viral load were successfully sequenced and the HDV viral load ranged from 2.27 × 10^5^ to 9.67 × 10^6^ copies/mL (Table 1). The seroprevalence of HBsAg were reported before as 8.4% in blood donors, 6.7% in HIV co-infected and 43.3% in CLD patients [14] but the actual prevalence of HBsAg, in which anti-HDV was currently screened, was 5.5%, 5.9% and 40.5% among the above respective groups (Fig. 1). The HBV DNA was detected with the median (IQR) viral load level of 3.39 log IU/mL (1.42–6.83) from 69.2% (18) anti-HDV positive sera samples. From 30.8% (8) anti-HDV positive cases, HBV DNA was undetected (Table 1). Among the six study subjects with HDV RNA sequenced, isolates ETH2170 and ETH2280 were included in the latter group but isolates; ETH3790, ETH4060, ETH4100 and ETH2056 showed HBV DNA viral load of 3.62 log IU/mL, 6.34 log IU/Ml, 3.15 log IU/mL and 8.39 log IU/mL, respectively (Table 1). Except isolate ETH2056 which was subgenotype D2, three of the above isolates were HBV subgenotype A1. All of the study subjects who were screened for anti-HDV antibody were characterized before for their anti-HCV status. However, none of the anti-HDV antibody positive study subjects were HCV PCR positive (Table 1).

### Phylogenetic analysis

**Fig. 2:**
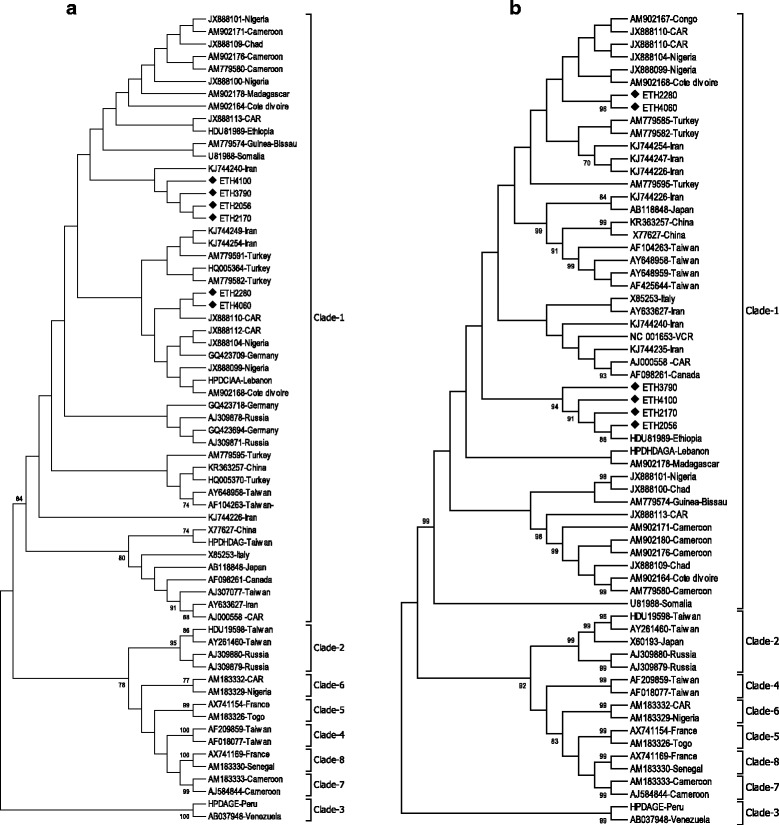
Phylogenetic analysis of HDV isolates based on a non-coding partial genome region (368 nt) (**a**) and full genome sequences (**b**). Reference strains were designated by their accession number followed by country of origin. The currently identified isolates indicated with initials ETH followed by four-digit numerical codes

The phylogenetic analysis from both non-coding partial genome region (368 nt) and the full genome sequences (~1678 nt) classified all the six isolates as HDV genotype 1 (HDV-1) (Fig. 2). The analysis of the sequences from the current study and reference sequences retrieved from the GenBank revealed that the current HDV-1 strains formed two monophyletic clusters in the phylogenetic analysis. The isolates ETH2170, ETH2056, ETH3790 and ETH4100 formed one independent cluster together with previously described single Ethiopian strain particularly in the full genome sequences analysis (Fig. 2b). The other two strains; ETH2280 and ETH4060 mostly clustered with West African strains (Fig. 2). Interestingly, this independent clustering was reproducible during the phylogenetic analysis of the full genome as well as the non-coding partial genome region sequences (Fig. 2a and b).

### Amino acid residues and nucleotides analysis

**Fig. 3:**
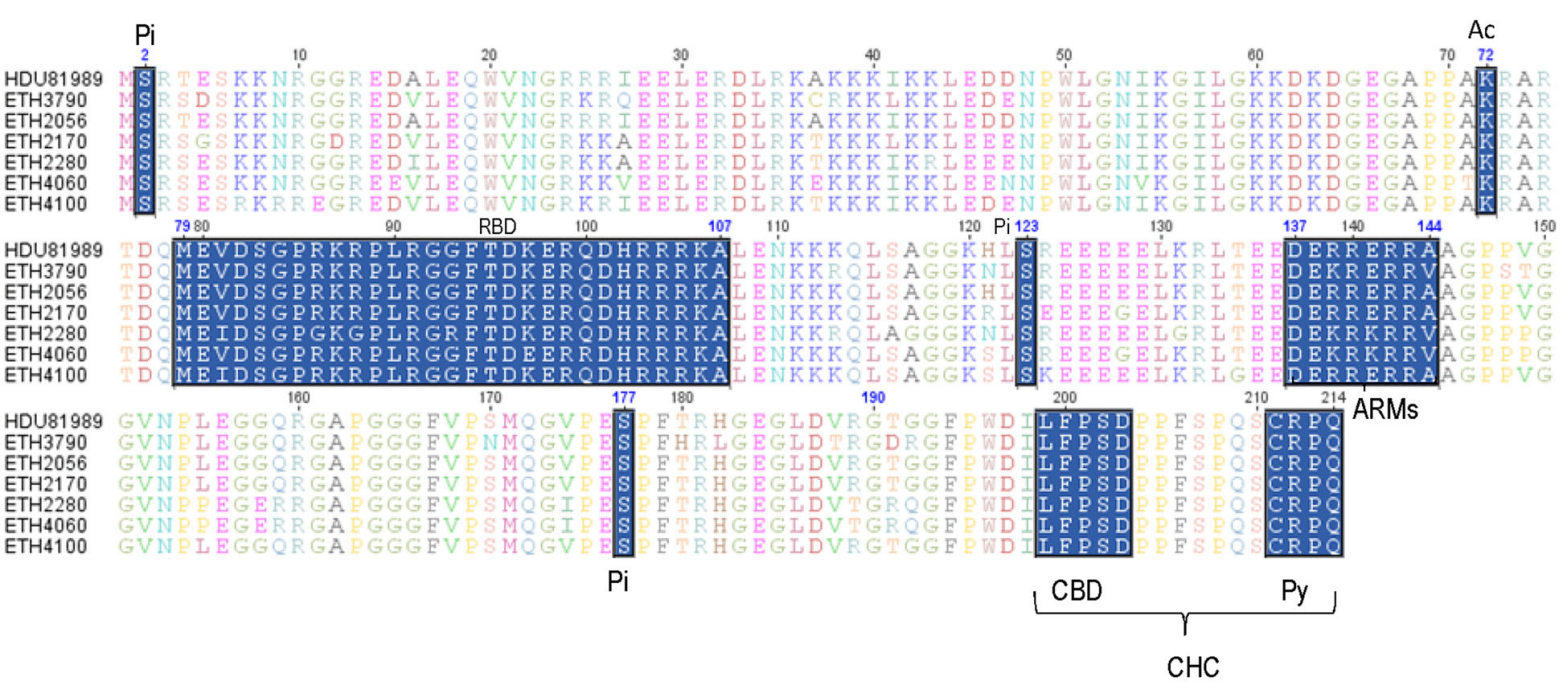
Alignment of amino acid sequences of the LHDAg depicting the genotypic, functional and immune epitope domains of the 19 C-terminal amino acids (196–214) of the clathrin heavy chain (CHC) interacting domain bearing a clathrin box-binding (CBD) domain (aa 199–203), a serine at aa 202, isoprenylation (Py) sites at 211–214. The figure shows phosphorylation sites (Pi) (aa 2, 123, 177), acetylation site (Ac) (aa 72), HDV RNA binding domain (RBD) (aa 79–107) and arginine-rich motifs (ARMs) (aa 137–144) of the sHDAg. Refrence sequence for alignment was made with Ethiopian HDV isolate (accession number: HDU81989)

**Fig. 4:**
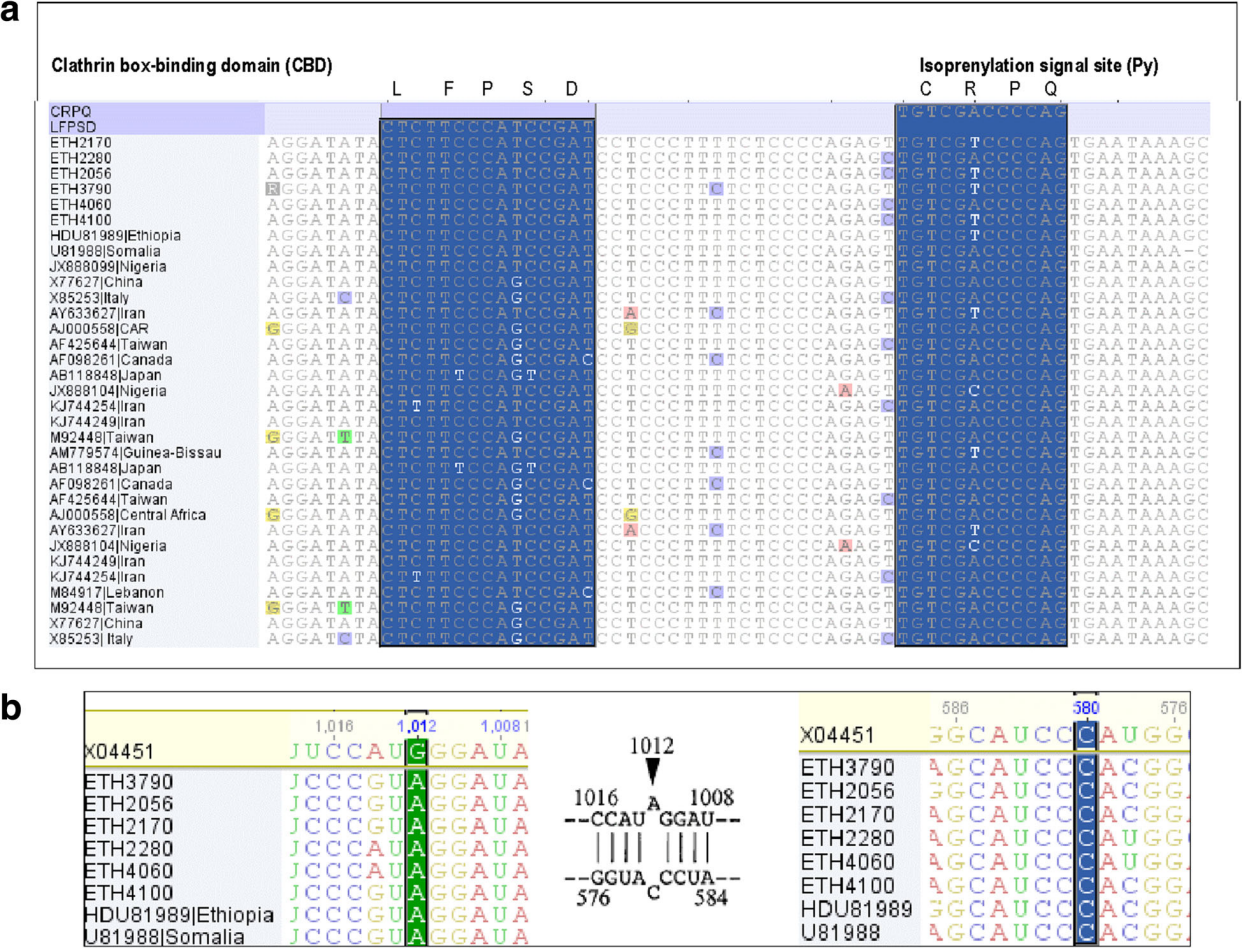
Alignment of the nucleotide sequences of the current isolates with the reference sequences from the GenBank that encode the carboxyl (C) terminal 19–20 residues of LHDAg showing the clathrin box-binding (CBD) domain and the isoprenylation signal (Py) site nucleotide substitutions variations (**a**), and the RNA editing targets of the anti-genomic HDV RNA (nt 1012) and its corresponding genomic RNA (nt 580) (**b**). The amino acid residues that constitute the CBD domain and the Py signal indicated above the nucleotide sequence in a single-letter amino acid abbreviation. Nucleotides positions of RNA editing targets are according to reference strain with accession number X04451

The CHC interacting domain at 19 C-terminal (^196^WDILFPSDPPFSPQS-CRPQ^214^) showed no amino acid variations in all of the six isolates. This includes the CBD (^199^LFPSD^203^) and the isoprenylation (Py) signal site (‘^211^CRPQ^214^’) (Fig. 3). All of the six isolates also showed serine (S) at amino acid position 202. The homology at nucleotide level in both CBD and Py regions were also compared with HDV-1 sequences retrieved from GenBank and showed high conservation with no nucleotide variations (Fig. 4a). Similarly, all of the isolates showed no nucleotide variations of the poly(A) signial domain sequences (946-CTTTATT-952) when compared to the refrecne strains of genotypes 1–3. The RNA editing targets of the anti-genomic HDV RNA (at nt 1012) and its corresponding genomic RNA (at nt 580) showed nucleotide A and C, respectively. However, unusual nucleotide changes (A to G) at nt 1014 were observed among four of the isolates (Fig. 4b). In the HDV-1 RNA editing domain of the sHDAg (aa 79–107), all of the six isolates showed amino acid changes only at two sites (aa 80 and 88) (Fig. 3). Moreover, the acetylation (AC) site (aa 72) revealed a lysine residue in all of the isolates. Similarly the amino acid compositions of the phosphorylation (Pi) sites (aa 2, 123, and 177) showed a serine residue in all the isolates. The domains of ARMs (aa 137–144) relatively showed little amino acid variations (Fig. 3).

## Discussion

HBV with a prevalence rate of 7.4–8.4% [14, 16] is hyperendemic in Ethiopia but data on HDV seroepedimiology and molecular diversity are generally lacking. This study, to the best of our knowledge, is the first description of HDV molecular characterization from a large study population comprising blood donors, liver disease patients and HIV infected individuals. The seroprevalence and viral detection rate of HDV observed among HBsAg positives cases in this study were unexpectedly low when compared to previous reports from Ethiopia [17, 18], other African countries and global data [7, 10, 19]. Nevertheless, reports showed an increasing incidence of global HDV infection due to dynamic population movement particularly in HIV-infected patients from HBV hyperendemic area [10]. For instance, in a European cohort of HIV (EuroSIDA), 14.5% seroprevalence of HDV with 87% viral RNA detection rate was reported [7]. Moreover, in areas of west and east African regions where geographical proximity and healthcare access were more or less similar with the current study setting, up to 70% of viral detection rate was reported [19–21].

In this study, however, there appeared to be low prevalence and/or high HDV viral clearance which could contradict with the notation that during multiple viral infections such as other hepatitis viruses and HIV, HDV is usually dominant and inhibits the others viruses [4, 22]. Especially, in immunodeficient patients, the HDAg was reported to stay longer because of the slow and weak immune response [23]. In contrast, more than 80% of anti-HDV antibody positive participants showed comparably high HBV DNA levels relative to reports elsewhere infected with HDV-1 genotype [4]. As the result, in particular HBV-HIV co-infected study population of the current study, it was necessary to examine the impact of more than a decade HIV ART exposure against HBV and consequent complex drug resistance and concomitant HBsAg variability profiles [14] on anti-HDV positivity and viremia rate. In this case, the variability of the surface protein might affect the degree of the antigenicity which potentially plays down HDV assembly and infectivity. It is known that certain amino acid sequences in the C-terminal domain of the surface protein are essential for the assembly of HDV particles [24]. In the current HIV infected study subjects, the prevalence of YM(I)DD motif HBV-drug resistant mutations i.e. rtM204V/I accounted for 29.3% and 15.5% rtL173V with concomitant immune escape HBsAg mutants of 13.8% rtM204V/sI195M, 3.4%rtM204I/sW196* and 10.3% rtV173L/rtL180M/rtM204V + sE164D /sI195M [14]. Except one case (PCR negative for HDV), none of the HBV-HIV co-infected patients who were characterized by these concomitant drug and surface gene mutants were positive for anti-HDV antibody. The absence of anti-HDV positivity in this group, therefore, supports the above speculation and previous findings [7, 24, 25], although the HDV seroprevalence data of the current study did not consider HBsAg negatives due occult HBV infections and /or anti-HBc positive cases. For instance, the immune escape HBsAg mutations such sQ164A and sE164D with concomitant rtV173L were reported to reduce the antigenicity of HDV particles [25]. Moreover, the HBV drug resistant mutations (rtM204V/I) are associated with changes in the overlapping envelope gene products, in particular, the gene encoding small envelope protein (s) at sI195M or sW196L/S/* [24] which in turn can impair HDV replication by compromising viral particles assembly and secretion [7, 24].

The blood donors and CLD patients of the current study were characterized by relatively less frequency of immune escape HBsAg mutations than their HIV counterparts (but higher than similar reports elsewhere) [14] may also partly explains the unexpectedly low rate of HDV viral detection. A more limited production of HBsAg by HBV genotype A than D [7] coupled with the above scenario might contribute to explain this observation since genotype A is the predominant isolate in Ethiopia [26]. Moreover, except one case, three of the strains in the current study were isolated from patients with HBV genotype A unlike previous studies that reported HDV-1 was mostly associated with HBV genotype D [4, 27]. However, in half of the HDV-1 isolates sequenced, HBV virus was suppressed to undetectable levels and thus co-genotyping of HBV was not possible and made the above explanation partial.

In this study, the phylogenetic analysis showed that all the six isolates were HDV-1 clustered into two monophyletic clusters and one of the clusters showed high relation to a previously described single strain from an Ethiopian patient isolated in Sweden [28]. Interestingly, this apparent segregation and independent clustering was reproducible when both the full genome and non-coding partial genome region sequences (368 nt) were considered for the phylogenetic analysis. Although HDV genotyping studies so far reported are very few [9, 13], analysis of HDV sequences from African countries showed the highest diversity of genotypes 1 and 5–8 [29, 30]. However, complete presence of HDV-1 from the current and previous studies in Ethiopia and neighboring Somalia [28] suggest that HDV-1 was a predominant clade occurring in Eastern African regions although majority of east African countries still uncovered.

The clade homogeneity observed in the phylogenetic analysis was also reflected from analysis of amino acid residues and nucleotide sequences of those important structural and functional domains of the last 19 C-terminal of LHDAg [2, 31, 32]. Among the essential sequences for HDV replication and maturation, the sequence encoding the C-terminal peptide of LHDAg of genotypes HDV1–8 are with the highest diversity compared to other regions of protein-coding sequences [32]. HDV-1 in which the current strains belongs was characterized by ^196^WDILFPSDPPFSPQS-CRPQ^214^ amino acid sequences of the 19-C terminal protein CHC domain [32] with CBD amino acid sequence of ‘LFP (A, S, V) D’ located at amino acids 199–203 [2]. With no significant nucleotide substitution variations, all the current isolates together with previously identified Ethiopian strain [28] showed the ^196^WDILFPSDPPFSPQS-CRPQ^214^ amino acid sequences with the CBD sequences of ^199^LFPSD^203^. Moreover, at aa position 202, they also exhibited a serine in contrast to Eurasian strains (which have an alanine residue) [4]. At the C-terminus of LHDAg which acts as a signal of isoprenylation for viron assembly and secretion are also characterized by amino acid sequence variations of ^211^CRPQ^214^, ^211^CTPQ^214^, and ^211^CTQQ^214^ in various HDV genotypes. Similarly, the HDV-1 of the current study showed ^211^CRPQ^214^ with four out of the six isolates showing a nucleotide change from A to T at the Py signal site but the CRPQ amino acid sequences were unaffected. This further proved absence of a failure in viron assembly and secretion due mutations of the Py signal of LHDAg [2]. The CHC domain sequences of the current study also showed absolute conservation at nucleotides level when compared with nucleotide sequences of the nearest HDV-1 strains retrieved from GenBank on the basis of the Basic Local Alignment Search Tool. Absence of significant amino acid and nucleotide sequences variation in the 19 C-terminal LHDAg showed HDV viral assembly and infectivity were unaffected and had a less likely association with the low rate of viral detection in this study. Moreover, the nucleotide constitutes of the RNA editing targets of the anti-genomic HDV RNA at nt 1012 and its corresponding genome site at nt 580 showed a known of tryptophan codon (TGG) which is required for efficient RNA editing and HDV replication [33, 34] instead of the amber stop codon (TAG).

The sHDAg amino acid sequences which have structural and functional roles were also compared among the six sequences. The amino acid sequence analysis of the post-translational phosphorylation site in the sHDAg protein showed serine at positions 2, 123 and 177 which is responsible for genomic HDV RNA synthesis (HDV antigenome RNA replication) [35] by interacting with cellular RNA polymeraseII [36]. Moreover, the acetylation site aa 72 showed a lysine residue in this study in which its substitution by an alanine re-localized the mutant sHDAg into the cytoplasm and associated with the diminished viral RNA accumulation and earlier LHDAg appearance [37, 38]. In contrast, although variation of amino acid sequence requirement for the RNA binding function may not be that much strict [28], three of the six isolates showed variations only on two amino acids positions (V81I and R88G).

## Conclusions

Exclusion of anti-HDV seroprevalence report from HBsAg negatives as the result of occult HBV infections and /or anti-HBc positive cases was the limitation of the current study. Despite a large study population screened in this study and hyperendemicity and genetic heterogeneity of HBV infection in Ethiopia, anti-HDV seroprevalence and HDV viremia rates were relatively low and even absent in those HBV-HIV co-infected with YM(I)DD motif drug selected HBsAg mutant variants. Thus, the potential role of HBV drug resistance associated polymerase mutations and concomitant surface protein variability on HDV viral assembly and infectivity needs further investigation. In contrast, the phylogenetic analysis showed the circulation of HDV-1 with the highest clade homogeneity and conservation of nucleotides and amino acid sequences on important structural and functional domains of HDV. This study provides new insights into the genetic diversity and molecular epidemiology of HDV-1 in Ethiopia where access to healthcare for viral hepatitis is totally absent.

## Abbreviations

3TC: Lamivudine
aa: Amino acids
Ac: Acetylation sites
ARMs: Arginine-rich motifs
CBD: Clathrin box-binding domain
CHC: Clathrin heavy chain
CLD: Chronic liver disease
HBsAg: Hepatitis B surface antigen
HBV: Hepatitis B virus
HCC: Hepatocellular carcinoma
HDAg: Hepatitis delta antigens
HDV-1: HDV genotype 1
HIV: Human immunodeficiency virus
IgG-Ab: Anti-HDV antibody
LC: Liver cirrhosis
PCR: Polymerase chain reaction
Pi: Phosphorylation site
Py: Isoprenylation signal site
RT: Reverse transcription
YM(I)DD: Tyrosine-methionine/isoleucine-aspartate-aspartate
